# Impact of Spirulina Extract-Loaded Quinoa Protein Isolate Nanoparticles on the Quality and Stability of Functional Set Yoghurt

**DOI:** 10.1007/s11130-025-01437-1

**Published:** 2025-11-28

**Authors:** Enass M. Abd El Maged, Hassan A. Elhendy, Samir M. Ahmed, Amira M. G. Darwish, Hesham Ali El-Enshasy, Tarek N. Soliman

**Affiliations:** 1 Home Economics Department, Faculty of Agriculture, Alexandria University, Alexandria, Egypt; 2 Food Industry Technology Program, Faculty of Industrial and Energy Technology, Borg Al Arab Technological University (BATU), Alexandria, Egypt; 3 Food Technology Department, Arid Lands Cultivation Research Institute (ALCRI), City of Scientific Research and Technological Applications (SRTA-City), New Borg El Arab, P.O. Box 21934, Alexandria, Egypt; 4 Innovation Centre in Agritechnology for Advanced Bioprocessing (ICA), Universiti Teknologi Malaysia (UTM), Johor, Malaysia; 5 Faculty of Chemical and Energy Engineering, Universiti Teknologi Malaysia (UTM), Johor Bahru, Johor, Malaysia; 6 City of Scientific Research and Technology Application (SRTA-City), New Burg Al Arab, Alexandria, Egypt; 7 Department of Dairy, Food Industries and Nutrition Research Division, National Research Centre, Dokki, Cairo, Egypt

**Keywords:** Spirulina extract, Quinoa protein isolate nanoparticles, Functional set yoghurt, Antioxidant potentials, Sensory acceptability

## Abstract

Protein and active compounds play a key role in maintaining human health as functional nutrients and as tools in structuring enhanced food products quality. This study explored the potentials of novel quinoa protein isolate nanoparticles (QPI-NPS) loaded with spirulina ethanol extract (SE) with different concentrations (0.2, 0.4, and 0.6%), and its impact on enhanced properties assessing; chemical, physical, functional and sensory attributes of set yoghurt on time intervals up to 21 days of cold storage. Characterization of the (QPI-NPS) using dynamic light scattering (DLS) revealed particle sizes ranging from 219.90 to 720.40 nm and the polydispersity indices (PDI) was between 0.270 and 0.386, with transmission electron microscopy (TEM) defining nanoparticle microstructure formation. Encapsulation efficiency reached a maximum of 89.75%. High-Performance Liquid Chromatography (HPLC) analysis identified gallic acid, chlorogenic acid, catechin, methyl gallate, naringenin, and quercetin as dominant phenolic compounds in SE and quinoa flour (QF). Results indicated that incorporating 0.4% spirulina extract quinoa protein isolate nanoparticles (SE-QPI-NPS) optimized particle size, stability, homogeneity, and encapsulation efficiency. The QPI-NPS fortification resulted enhanced the fortified yoghurt chemical, viscosity, antioxidant potentials and sensory attributes. This study highlights the promising applications of novel QPI-NPS as a delivery vehicle for spirulina extract, improving the functionality and consumer acceptability of functional food products.

**Supplementary Information:**

The online version contains supplementary material available at 10.1007/s11130-025-01437-1.

## Introduction

The incorporation of spirulina extract-loaded quinoa protein isolate nanoparticles into functional set yoghurt represents a promising advancement in the field of food science, particularly in enhancing the nutritional quality and stability of dairy products. Spirulina, a blue-green microalga, is recognized for its rich nutritional profile, including high protein content, essential fatty acids, vitamins, and antioxidants, which contribute to its potential as a functional food ingredient [1, 2]. The growing consumer demand for health-promoting foods has prompted researchers to explore innovative methods of fortifying dairy products, such as yoghurt, with bioactive compounds derived from Spirulina. Studies have demonstrated that the addition of Spirulina can enhance the growth of beneficial bacteria, such as Bifidobacterium and Lactobacillus, while simultaneously improving the oxidative stability and overall quality [2, 3].

Many plant-based sources nutrients show lower bioavailability compared to animal sources. Reduced absorption is related to ease of binding to inhibitors (*e.g*., polyphenols, and tannins), Maintaining appropriate nutrients levels is essential for protein synthesis, energy transport and storage, oxygen transport, and many other metabolic functions [2, 3]. The potential health benefits associated with the consumption of Spirulina-enriched yoghurt extend beyond mere nutritional enhancement. Studies indicate that Spirulina possesses antioxidant, anti-inflammatory, and immunomodulatory properties, which can contribute to the prevention of chronic diseases [1, 4]. The incorporation of such functional ingredients into yoghurt aligns with the increasing trend toward functional foods that support health and wellness. Moreover, the innovative approach of using nanoparticles for encapsulation addresses challenges related to the stability and bioactivity of sensitive compounds, thereby ensuring that the health benefits are retained throughout the product's shelf life [5].

Nanotechnology plays a crucial role in this context, as the encapsulation of spirulina extracts in quinoa protein isolate nanoparticles can enhance the bioavailability and stability of the bioactive compounds during storage and digestion [5]. The physicochemical properties of these nanoparticles, including their size, morphology, and encapsulation efficiency, are critical factors that influence their effectiveness as delivery systems for functional ingredients [5]. Research has shown that encapsulated Spirulina exhibits improved sensory attributes and antioxidant activity compared to non-encapsulated forms, which is essential for consumer acceptance and product quality [6, 7]. Furthermore, the use of quinoa protein isolate as a matrix for nanoparticle formation not only provides a plant-based protein source but also contributes to the functional properties of the yoghurt, enhancing its nutritional profile [3].

This study aimed to apply novel quinoa protein isolate nanoparticles (QPI-Ns) loaded with spirulina extract, for the fortification of functional set yoghurt and its functionality assessment compared with plain yoghurt as control and two fortified yoghurt samples with the free forms of spirulina powder and spirulina extract; in order to evaluate the impact of nanoencapsulation technique on chemical, functional (antioxidant potentials), physical (viscosity) and consumer acceptability, towards favorable health-promoting food products.

## Materials and Methods

Materials and Methods are detailed in the Supplementary Material.

### Preparation Quinoa Flour and Protein Isolate (QF & PI)

Quinoa seeds were prepared according to [3]. For quinoa protein isolate (QPI) preparation; the quinoa flour was defatted according to [3]. Quinoa protein isolate was prepared according to [3] as illustrated in (Fig. SM1).

### Spirulina Extraction (SE)

Spirulina ethanol extract was obtained using 80% ethanol according to [8].

### Phenolic Compounds Profile (HPLC)

High-performance liquid chromatography (HPLC) was performed using an Agilent 1260 system to analyze the samples according to [8].

### Spirulina Extract Encapsulation

The quinoa protein isolate (QPI) solution was prepared as described by [9]. Subsequently, different concentrations of spirulina extract (SE) 0.2, 0.4, and 0.6% (w/v) were incorporated into the protein solution, with protein–polyphenol ratios of 1:0.2, 1:0.4, and 1:0.6, respectively. The same procedure was used to prepare a control sample, a QPI solution without SE [10].

### Characterization of QPI-NPS by DLS

The QPI, QPI-SE 0.2, QPI-SE 0.4, and QPI-SE 0.6 nanoparticles, were appropriately diluted and subjected to dynamic light scattering (DLS) analysis using a Nano-ZS90 instrument (Malvern, UK) to determine their respective particle size, polydispersity index (PDI), and zeta potential. The experiments were carried out in triplicate. Before determination, every sample of nano micelle was brought to an equilibrium state at a temperature of 25 ◦C [10].

### Encapsulation Efficiency (EE)

The encapsulation efficiency of SPE-QPI nanoparticles and regression analysis established a calibration curve using pure gallic acid at varying concentrations ranging from 0.155 to 0.180 mg/mL. The obtained regression coefficient was 0.9989 according to [10].

### Microstructural Analysis via Transmission Electron Microscopy (TEM)

The microstructure of the nanoparticles was examined using a JEOL JEM-1400 plus TEM, operating at 100 kV, with a magnification level of 200,000 × employed for imaging [10].

### Preparation of Functional Set Yoghurt

Raw buffalo milk was utilized to prepare functional set yoghurt according to [10]. The milk was divided into four groups: a control group (C) consisted of plain yoghurt, and three treatment groups designated as Treatment 1 (T1) was fortified with free spirulina extract (SE) at a concentration of 400 mg, Treatment 2 (T2) was fortified with 2,000 mg of spirulina powder, and Treatment 3 (T3) was enhanced with 1400 mg of nano-encapsulated SE, specifically utilizing 0.4 SE-QPI nanoparticles (NPs). The selection of the 0.4 SE-QPI NPs formulation was based on the results of characterization studies that evaluated the polydispersity index, particle size, surface charge, and encapsulation efficiency (refer to Table 3). The prepared yoghurts were stored at a temperature of 5 ± 1 °C, in preparation for subsequent analyses.

### Physicochemical Properties of Set Yoghurt

The pH of the yoghurt products was measured using a pH meter (model IQ 240, I.Q. Scientific Instruments Inc., San Diego, CA) equipped with automatic temperature compensation (A.T.C.) probe. Acidity was assessed following the [10, 11] guidelines.

### Evaluation of Antioxidant Potentials

The sample capacity to scavenge free radicals was evaluated using DPPH (2,2-diphenyl-1-picrylhydrazyl) as described by [10, 12]. The total phenolic content of both the SE and the encapsulated SEE was assessed using the Folin-Ciocalteu method[10]. The total flavonoid content in the extracts was determined following the method established by [12].

### Viscosity

The Bohlin coaxial cylinder viscometer (Bohlin Instrument Inc., Sweden) was connected with V88 viscometer programming software to assess the apparent viscosity of fresh yoghurt and at storage period of 21 days. The viscosity readings were recorded at 20 ± 2 °C in the upward move across shear rates from 19 to 1236 S-1 [13].

### Sensory Evaluation

In a controlled environment, sensory attributes of yoghurt were evaluated by 18 panelists aged between 27 to 51 years, from the Dairy Department, Food Industries and Nutrition Research Institute, the National Research Centre. The criteria for selection depended on their experience related to yoghurt products, and they were instructed to rinse their mouths between samples. The evaluation was carried out as described by [13].

### Statistical Analysis

All data are presented as mean ± standard deviation (SD). Statistical analyses were conducted using one-way analysis of variance (ANOVA) followed by Duncan’s test applying Co-Stat software (version 8). Statistical significance between groups was established at *p* ≤ 0.05. Data visualization was performed using GraphPad PRISM (Version 8.0.1, GraphPad Software, San Diego, CA, USA).

## Results and Discussion

### Determination of Phenolic Compounds (HPLC)

Results indicated that quinoa and spirulina showed to differ in the types and amounts in phenolic compounds. By comparing compounds' retention periods to those of genuine standards examined under the same circumstances, the results (Table SM1) showed that SAP showed significant content of gallic acid, chlorogenic acid, catechin, methyl gallate, naringenin, quercetin (201.84, 138.73, 41.62, 21.44, 31.15, 29.92 µg/g), while QF showed that gallic acid, chlorogenic acid, naringeninn, cinnamic acid (25.98, 165.64, 39.39, 50.06 µg/g) were the dominant polyphenol compounds. These bioactive phenolic compounds are found to act as antioxidants, making spirulina and quinoa a potential candidate for developing as natural preservatives in food and medicine [14]. Obtained results revealed that both quinoa and spirulina offer a good content of phenolic compounds that can complement each other, enhancing the antioxidant effect, which were supported by antioxidant potential results.

### Characterization of QPI-NPS

(Table 1) presents the characteristics of QPI nanoparticles and SE-loaded QPI-NPS. The average particle size of unloaded QPI-NPS was 314.70 ± 67 nm. Encapsulating spirulina ethanol extract in QPI-NPS showed significant increase in particle size up to 479.30 ± 29 nm, 720.40 ± 216 nm in 0.4 and 0.6 QPI-NPS, respectively. This suggests that at lower concentrations, the extract might be better integrated within the nanoparticles, leading to smaller sizes. At higher concentrations, the extract might be causing aggregation, hence the larger size. Similar observation reported by [15]. The zeta potential of unloaded QPI-NPS exhibited −14.6 ± 3 mV (Table 1) and (Fig. SM2). The incorporation of SE different concentrations 0.2, 0.4, and 0.6 QPI-NPS led to a more negative zeta potential, ranging from −24.3 ± 4 mV to −25.6 ± 5 mV with 0.4 SE-QPI NPs. This significant increase in negative charge suggests improved stability of the nanoparticles due to enhanced electrostatic repulsion between particles, potentially preventing aggregation. However, the negative charge decreased with 0.6 SE-QPI NPs to −24.7 ± 4 mv. The observed phenomenon may be attributed to the capacity of polyphenolic extracts to modify the secondary structure of the QPI [10]. Moreover, the significant rise in electro-negativity showed to be aligned with the increase in SE which implies that the electrostatic repulsion of the droplets is enhanced when SE polyphenolics form a complex with QPI. The polydispersity index of unloaded QPI-NPS was 0.464, indicating a moderately heterogeneous size distribution. While, the PDI (polydispersity index) values for SE-loaded QPI-NPS ranged from 0.270 to 0.386, suggesting more homogeneous size distribution compared to unloaded nanoparticles. Lower PDI is generally desirable for nanoparticle systems as it indicates greater uniformity.

**Table 1:** Nanoparticles (QPI-NPS) characterization of spirulina extract (SE) loaded in 1 g quinoa protein isolate nanoparticles (QPI-NPs) (0.2, 0.4, 0.6)

Sample	Size (nm)	Zeta potential (mV)	PDI	Encapsulation efficiency (%)
QPI NPs	314.70 ± 67^c^	−14.6 ± 3^a^	0.464^a^	-
0.2 SE-QPI NPs	219.90 ± 56^d^	−24.3 ± 4^b^	0.313^b^	89.75 ± 1.17^a^
0.4 SE-QPI NPs	479.30 ± 29^b^	−25.6 ± 5^c^	0.270^c^	87.95 ± 1.05^a^
0.6 SE-QPI NPs	720.40 ± 216^a^	−24.7 ± 4^b^	0.386^b^	80.12 ± 2.12^a^

Values are represented as means ± SD, Values in the same row with different superscript letters are significantly different (*P*<0.05), *PDI* Polydispersity index, *EE* Encapsulation efficiency, *QPI NPs* quinoa protein isolate nanoparticles, *0.2 SE-QPI NPs *0.2 g spirulina extract loaded in 1 g quinoa protein isolate nanoparticles, *0.4 SE-QPI NPs* 0.4 g spirulina extract loaded in 1 g quinoa protein isolate nanoparticles, *0.6 SE-QPI NPs *0.6 g spirulina extract loaded in 1 g quinoa protein isolate nanoparticles.

Table (1) showed that the EE insignificantly decreased with increasing extract concentration, potentially due to saturation of the nanoparticles. The encapsulation efficiency of spirulina extract within QPI-NPS ranged from 80.12 ± 2.12% to 89.75 ± 1.17%. This signifies the successful entrapment of the extract within the nanoparticles.

These results align with earlier research by [16] regarding the particle size and PDI of flavonoids in QPI. The obtained results indicated that encapsulating spirulina extract 0.4% within QPI-NPS can be achieved with good efficiency and appears more stable, homogenous and decreased PDI. This phenomenon may be advantageous in terms of stability for protein–polyphenol complexes in a wide range of food applications [10]

### Microstructural of SE-QPI-NPS Using Transmission Electron Microscopy

**Fig. 1:**
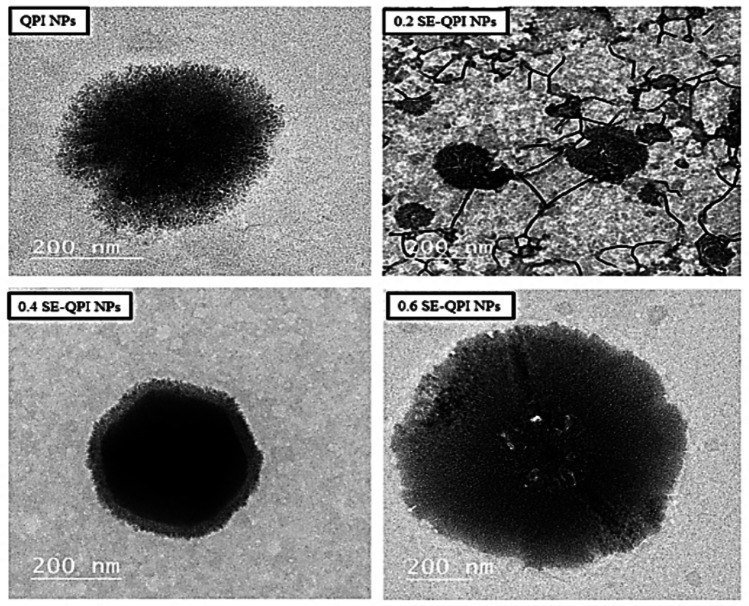
Transmissions electron microscopy (TEM) of quinoa protein isolates nanoparticles.* QPI NPs* quinoa protein isolate nanoparticles, *0.2 SE-QPI NPs* 0.2 g spirulina extract loaded in 1 g quinoa protein isolate nanoparticles, *0.4 SE-QPI NPs* 0.4 g spirulina extract loaded in 1 g quinoa protein isolate nanoparticles, *0.6 SE-QPI NPs* 0.6 g spirulina extract loaded in 1 g quinoa protein isolate nanoparticles

The microstructural analysis of spirulina extract (SE) loaded quinoa protein isolate nanoparticles (QPI-NPS) using transmission electron microscopy (TEM) illustrated in (Fig. 1) provides insights into the morphology and structure of these nanoparticles. TEM images are crucial for understanding the morphology of nanoparticles, which can impact their stability, encapsulation efficiency, and release characteristics. The QPI-NPS refers to small and spherical particles that often indicate uniform distribution and high ability to encapsulate bioactive ingredients [17]. The quinoa protein nanoparticles can enhance the stability and bioavailability of spirulina compounds in food applications [18]. The particle size increased according to the concentration of spirulina extract loaded into QPI, which influences their microstructure. At lower spirulina extract loading (0.2 g SE *per* 1 g QPI); the nanoparticles maintained their spherical morphology, with a slight increase in particle size compared to unloaded QPI NPs. The particles remained mostly discrete and well separated, suggesting efficient encapsulation and minimal aggregation. While at the highest loading level (0.6 g SE *per *1 g QPI), the TEM images showed a further increase in particle size and the appearance of more irregular and less defined shapes. These results agree with particle-sizer DLS results [19].

### Characterization of Fortified Functional Set Yoghurt

The chemical composition of functional set yoghurt fortified with (SE) and SE encapsulated in (QPI-NPS) (Table 2), highlights significant differences in total solids, fat percentage, total protein, and ash content among the control and fortified samples.

**Table 2:** Chemical composition of fortified functional set yoghurt

Sample	Total solids	Fat%	Total Protein %	Ash
C	13.88 ± 0.30^c^	4.30 ± 0.1^a^	3.85 ± 0.02^c^	0.75 ± 0.01^a^
T1	14.28 ± 0.09^c^	4.30 ± 0.05^a^	4.04 ± 0.04^b^	0.76 ± 0.01^a^
T2	15.61 ± 0.10^a^	4.50 ± 0.0^a^	4.87 ± 0.05^a^	0.79 ± 0.02^a^
T3	15.28 ± 0.12^b^	4.20 ± 0.05^a^	4.80 ± 0.02^ab^	0.77 ± 0.01^a^

Values are represented as means ± SD

Values in the same row with different superscript letters are significantly different (*P*<0.05).

*C* control plain set yoghurt prepared from buffalo milk, *T1* yoghurt fortified with free spirulina extract (SE) at a concentration of 400 mg, *T2* yoghurt fortified with 2 g spirulina powder, *T3* yoghurt fortified with 0.4 g spirulina extract nano encapsulated which containing 400 mg spirulina extract loaded in 1,000 mg quinoa protein isolate.

The control yoghurt has a total solid content (TS) of 13.88%, while the fortified samples exhibit significant increase in total solids; while T2 showed the highest TS (15.61%) which can be attributed to the high nutritional density of spirulina, that is known for its rich protein and mineral content [20]. The total protein reflects this enhancement, as T2 achieved 4.87%, significantly higher than the control's 3.85%. This suggests that the incorporation of spirulina enhanced the protein content and improved the nutritional quality of the fortified yoghurt [21]. Despite the growing evidence of the benefits of the plant-based diet, challenges in misconceptions about nutrient adequacy can prevent individuals from depending on plant-based eating. Applying modern techniques and proper design of plant-based diets can meet the nutritional requirements and offer a sustainable approach to disease prevention. This indicates the importance of bridging the gap between scientific evidence and practical applications in functional foods [21]. In terms of fat content, the samples showed insignificant differences, while T2 showed insignificant increase to 4.50%. This stability in fat content indicates that the fortification process does not adversely affect the yoghurt texture and mouthfeel, which are aligned with sensory evaluation (Fig. 3) [22]. The ash content, which is an indication of the mineral content, also insignificantly increased in the fortified samples, particularly in T2 and T3 suggesting that the addition of spirulina contributes to enhanced mineral content of the fortified yoghurt, which agreed with what previously reported [23]

The encapsulation technique of spirulina extract in QPI-NPS enhances the stability and bioavailability of the bioactive compounds present in spirulina, potentially leading to improved health benefits [24]. The encapsulation process can protect sensitive compounds from degradation during processing and storage, thereby maintaining their functional properties [25]. Previous study reported that encapsulated bioactive compounds can exhibit enhanced solubility and stability, which is essential for their effectiveness in food applications [25]. The fortification of yoghurt with spirulina extract, both in free form and encapsulated form in QPI-NPS, significantly enhances its chemical composition, particularly in terms of total solids and protein. These changes improve the nutritional value of the fortified yoghurt and contribute to its functional properties, making it a good option for health-conscious consumers. These findings underscore the potential of using spirulina and quinoa protein isolates as effective ingredients in the development of functional dairy products.

### pH and Acidity

Fig. (SM2) illustrates the pH and acidity of the functional set yoghurt fortified with free (SE) and SE encapsulated in QPI-NPs along the 21 days of storage. The pH values decreased over the time for all the samples, indicating an increase in acidity due to the metabolic activities of the starter cultures and the conversion of lactose into lactic acid [26] The significant decrease in pH is more pronounced in samples with higher concentrations of spirulina, such as T2, which had the lowest pH at the end of the storage period. This may be due to the stimulation of lactic acid bacteria, similar to how quinoa flour enhances probiotic viability [26].

The nanoencapsulation of spirulina extract in QPI-NPs (T3) results in pH and acidity values were similar to the control, then T1 and T2 suggesting stabilized fermentation dynamics. This could indicate that nanoencapsulation controls the impact of spirulina on pH and acidity changes during storage. Overall, the addition of spirulina, especially in its free or powdered form, tends to enhance the acidity of yoghurt, which is consistent with findings that spirulina can stimulate the growth of lactic acid bacteria [6]. However, the nanoencapsulation method may moderate these effects, potentially offering a way to stabilize the product's properties during storage.

### Antioxidant Properties

The antioxidant properties of functional set yoghurt fortified with spirulina are significantly enhanced by the fortification with spirulina (Table 3). Spirulina, particularly in its nanoencapsulated form (T3), significantly enhanced the antioxidant activity of yoghurt compared to the control. This is consistent with spirulina known antioxidant properties, which include high levels of phenolic compounds, carotenoids, and phycocyanin [27]. The nanoencapsulation of spirulina extract (T3) resulted in significant increase in the total phenolic content to show the highest DPPH antioxidant activity among the samples. This suggests that nanoencapsulation may protect and enhance the bioactive compounds in spirulina, maintaining higher antioxidant levels over time [28]. On the other hand, free spirulina powder did not significantly affect the antioxidant activity compared to the control, as its effect was less pronounced (T1). This might be due to the differences in bioavailability or stability of the bioactive compounds in powder free form [6]. The significant differences in antioxidant activity and phenolic content indicating that the addition of spirulina, particularly in nanoencapsulated form, showed an observed impact on the antioxidant properties of yoghurt. Overall, the fortification with spirulina enhanced antioxidant properties, with nanoencapsulation offering a potentially recommended method for maintaining these benefits over time. Spirulina’s high content of antioxidants such as beta-carotene, phycocyanin, and phenolic compounds was reported to contribute to its ability to enhance the antioxidant capacity of yoghurt [27, 28]. Consuming plant-based diets, showed to exhibit lower rates of chronic diseases and longer life. Rich diets in antioxidants and phytochemicals, can contribute to protective effects such as; lower blood pressure, improved lipid profiles, and reduced the risk of coronary artery disease [6].

**Table 3:** Antioxidant activity and total phenolic/flavonoid content of functional set yoghurt

Samples	Total phenolic content (mg GAE/g)				DPPH (%)			
	Fresh	7 days	14 days	21 days	Fresh	7 days	14 days	21 days
C	27.52^d^ ± 3.88	35.80^c^ ± 1.25	41.25^d^ ± 3.81	39.09^d^ ± 4.32	21.55^d^ ± 2.05	30.02^d^ ± 3.52	32.01^d^ ± 4.35	30.43^d^ ± 4.54
T1	59.22^b^ ± 2.50	76.15^b^ ± 4.37	79.38^b^ ± 4.92	76.38^b^ ± 3.65	48.27^b^ ± 4.34	49.24^b^ ± 3.51	51.21^b^ ± 4.65	49.15^b^ ± 5.26
T2	34.95^c^ ± 0.49	52.36^c^ ± 5.01	55.75^c^ ± 9.05	52.54^c^ ± 7.67	35.52^c^ ± 5.35	37.85^c^ ± 2.47	39.12^c^ ± 5.23	36.45^c^ ± 7.24
T3	77.56^a^ ± 2.15	89.92^a^ ± 8.12	95.66^a^ ± 5.21	96.12^a^ ± 6.73	53.66^a^ ± 5.94	55.24^a^ ± 3.54	57.77^a^ ± 5.57	55.18^a^ ± 4.35

Values are represented as means±SD, Values in the same row with different superscript letters are significantly different (*P*<0.05), *C* control plain set yoghurt prepared from buffalo milk, *T1* yoghurt fortified with free spirulina extract (SE) at a concentration of 400 mg, *T2* yoghurt fortified with 2 g spirulina powder, *T3* yoghurt fortified with 0.4 g spirulina extract nano encapsulated which containing 400 mg spirulina extract loaded in 1,000 mg quinoa protein isolate.

### Viscosity

**Fig. 2:**
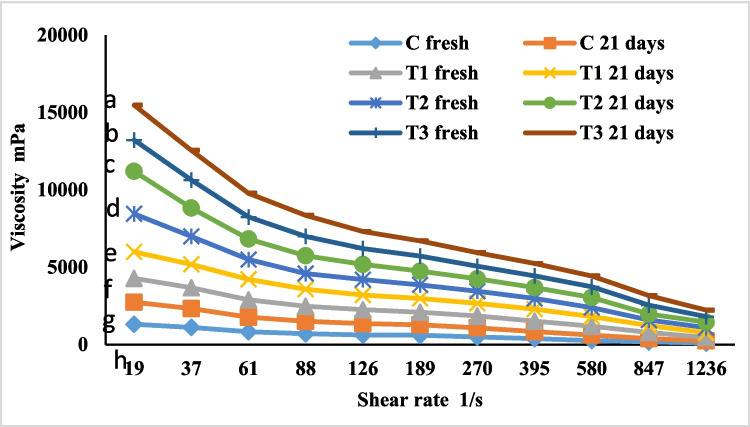
Viscosity of functional fortified set yoghurt. Values are represented as means. Lowercase letters indicate the significant differences between all samples at the same time interval (*P* < 0.05). *C* control plain set yoghurt prepared from buffalo milk, *T1* yoghurt fortified with free spirulina extract (SE) at a concentration of 400 mg, *T2 *yoghurt fortified with 2 g spirulina powder, *T3* yoghurt fortified with 0.4 g spirulina extract nano encapsulated which containing 400 mg spirulina extract loaded in 1000 mg quinoa protein isolate

The viscosity of the functional set yoghurt fortified with free spirulina extract (SE) and SE encapsulated in QPI-NPs varies significantly (*P* < 0.05) across different shear rates and storage times, as shown in (Fig. 2). Spirulina, in either free extract form (T1), powder form (T2), or nanoencapsulated (T3), enhanced the yoghurt viscosity compared to the control. This aligns with findings that spirulina can increase the viscosity of dairy products due to its protein and fibre content [6, 24]. The nanoencapsulation of SE (T3) resulted in a significant increase in viscosity compared to free spirulina extract (T1), suggesting that nanoencapsulation may stabilize the bioactive compounds and help maintain consistent viscosity over time. The highest viscosity is observed in yoghurt fortified with spirulina powder (T2), likely due to the higher concentration of spirulina in this treatment, which supplies more protein and fibre, enhancing the product's texture [6, 23]. The significant differences indicated that adding spirulina, in powder form, showed significant effect on yoghurt viscosity. Overall, incorporating spirulina into yoghurt improves viscosity. Nanoencapsulation provides a moderate viscosity increase while potentially stabilizing the product's properties over time.

### Sensory Evaluation of Functional Fortified Set Yoghurt

**Fig. 3:**
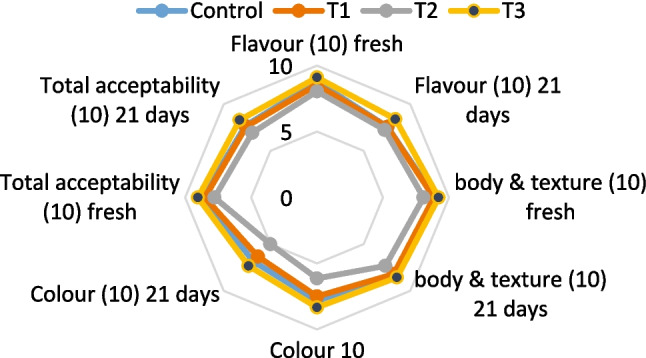
Sensory evaluation of fortified functional set yoghurt. Values are represented as means ± SD, *C* control plain set yoghurt prepared from buffalo milk, *T1* yoghurt fortified with free spirulina extract (SE) at a concentration of 400 mg, T2 yoghurt fortified with 2 g spirulina powder, *T3* yoghurt fortified with 0.4 g spirulina extract nano encapsulated which containing 400 mg spirulina extract loaded in 1,000 mg quinoa protein isolate

Functional fortified set yoghurt samples are represented in (Fig. SM3). The sensory evaluation of functional set yoghurt fortified with free spirulina extract (SE) and SE loaded-quinoa protein isolate nanoparticles (QPI-NPS) (Fig. 3), reveals the impacts spirulina fortification on the yoghurt 's flavor, body & texture, color, and overall acceptability. The fortification with spirulina affected significantly the sensory properties of yoghurt, with varying degrees of acceptance depending on the form of spirulina used [29].

Nanoencapsulation of spirulina extract (T3) appears to yield the best sensory outcomes. T3 gained the highest scores of flavors, body & texture, color, and total acceptability, suggesting that nanoencapsulation helps positively to preserve the desirable sensory attributes of yoghurt [29]. Yoghurt with spirulina powder (T2) received the lowest scores, indicating that this form of spirulina may negatively affect the sensory experience. This could be due to the color changes to green caused by the spirulina powder fortification (Fig. SM3). Same for T1 that showed yellowish color which panelists may find unappealing (Fig. SM3). The strong flavor of spirulina may also be a factor [30]. It is crucial to consider spirulina fortification impact on flavor and overall acceptability. Nanoencapsulation seems to mitigate the negative effects on flavor, as evidenced by the higher scores for T3 [30]. The decline in sensory scores over 21 days, was due to biochemical changes during storage. However, T3 maintained the highest scores, indicating better stability in sensory attributes [30]. In conclusion, nanoencapsulation of spirulina extract appears to introduce an effective method for fortifying yoghurt while enhancing its sensory quality [29]. Spirulina powder negative impact led to lower acceptability [6]

## Conclusion

In conclusion, the research presented underscores the potential of incorporating (QPI-NPS) into functional set yoghurt. The findings revealed that the fortification with spirulina enhanced the nutritional profile of yoghurt through increased protein, phenolic content and improved antioxidant properties, which helped in promoting health benefits. The spirulina extract within QPI-NPS demonstrated high encapsulation efficiency and stability, indicating that this method protects bioactive compounds during processing and storage. The physicochemical analysis exhibited favorable changes in total solids, protein content, and viscosity, which are essential for consumer acceptance and product quality. Furthermore, the sensory evaluation indicated that the nanoencapsulation mitigated the negative impact of spirulina on flavor and texture, resulting in higher overall acceptability compared. These findings suggest that the use of novel spirulina extract-loaded QPI-NPS enhanced the functional properties of yoghurt aligning with the increasing consumer demand for health-oriented food products. The study contributes to the application of nanotechnology technique in developing food products, that offer both nutritional and sensory benefits. Towards future research; in vivo validation can be recommended, evaluation of the long-term stability of these formulations in industrial applications and further exploration of health benefits for human consumption, paving the way for innovative solutions in the functional food industrial sector.

## Supplementary Information

Below is the link to the electronic supplementary material.

Supplementary file1 (DOCX 503 KB)

## Data Availability

The data is available under request.
