# Supplementary material for: Impact of Spirulina Extract-Loaded Quinoa Protein Isolate Nanoparticles on the Quality and Stability of Functional Set Yoghurt
Source: Plant Foods Hum Nutr. 2025 Nov 28;80(4):202. doi: 10.1007/s11130-025-01437-1 (PMC12662863; doi:10.1007/s11130-025-01437-1)
Supplement: Supplementary file 1 — Supplementary file1 (DOCX 503 KB) [file 11130_2025_1437_MOESM1_ESM.docx]

### **Supplementary Materials**

### Materials and methods

Sweet quinoa seeds Cv. Masr1 were obtained from Agricultural Research Center (ARC), Giza, Egypt. Spirulina powder was obtained from the culture collection of algae at Chad. Buffalo milk was obtained from Faculty of Agriculture, Cairo University, Giza, Egypt. Yoghurt starter (containing mixture of *Lactobacillus delbreukii* subsp *bulgaricus* and *streptococcus thermophilus* (1:1)) was obtained from Christen Hansen Laboratories, Copenhagen, Denmark, and kept at –18°C, until activated before use in sterilized reconstituted skim milk powder (SMP) obtained from Dairy America, Inc., California, USA, composed of 34% protein, 51% lactose, 1.2% fat, 8.2% minerals, and 4% moisture). Chemical materials used were purchased from Sigma Aldrich, Merck (Sigma Aldrich, Merck St. Louis, MO, USA). All reagents and solvents used were analytical grade.

**Preparation quinoa flour and protein isolate (QF & PI)**

Quinoa seeds were sieved 250-micrometer opening to foreign materials, soaked in water 12h/ 20^o^C, then washed till saponin removal and dried at 45∘C/12h [29]. Seeds then were dried to 12% moisture at 120°C/20 min, cooled at room temperature, grounded, sieved with a mesh size of 20.0 (840 μm), packed in polythene plastic bags and stored at -20 °C.

For quinoa protein isolate (QPI) preparation; the quinoa flour was defatted according to Folch [28]. Quinoa protein isolate was prepared according to El-Sohaimy [29]. Fifty grams of defatted quinoa flour were suspended in 1000 mL deionized distilled water (1:10 v/v), at pH 8.5, stirred for 1 h at 60oC/30 min, centrifuged at 3000 rpm at 15°C/ 30 min (model K241R, Pro-Research, Centurion Scientific Ltd, UK). The pH was acidified to 4.5, refrigerated at 4oC/1h for precipitation, centrifuged at 3000 rpm at 15°C/ 30 min. The protein isolate was the neutralized and freeze dried at -20oC.The obtained quinoa flour was defatted three times with chloroform: methanol (2:1), 1:10 w/v with shaking for 2 h according to Folch [28]. Quinoa protein isolate was prepared according to El-Sohaimy [29]. Fifty grams of defatted quinoa flour were suspended in 1000 mL deionized distilled water (1:10 v/v), and the pH was adjusted to 8.5 using 2N (NaOH) in order to facilitate quinoa protein solubility for higher yield of protein isolate. The suspension was stirred for 1 h while maintaining the pH at the determined value to reach the maximum level of solubility at 60^o^C/30 min. The mixture was centrifuged at 3000 rpm at 15°C/ 30 min by a high-speed cooling centrifuge (model K241R, Pro-Research, Centurion Scientific Ltd, UK). The supernatant was then adjusted to pH 4.5 with HCl 2N which is the isoelectric point of most proteins, refrigerated to 4^o^C/1h to facilitate protein precipitation and the centrifuged at 3000 rpm at 15°C/ 30 min for protein separation. The protein isolate was then neutralized with 2N (NaOH), freeze dried -20oC/ 5days as illustrated in (**Fig SM1).**


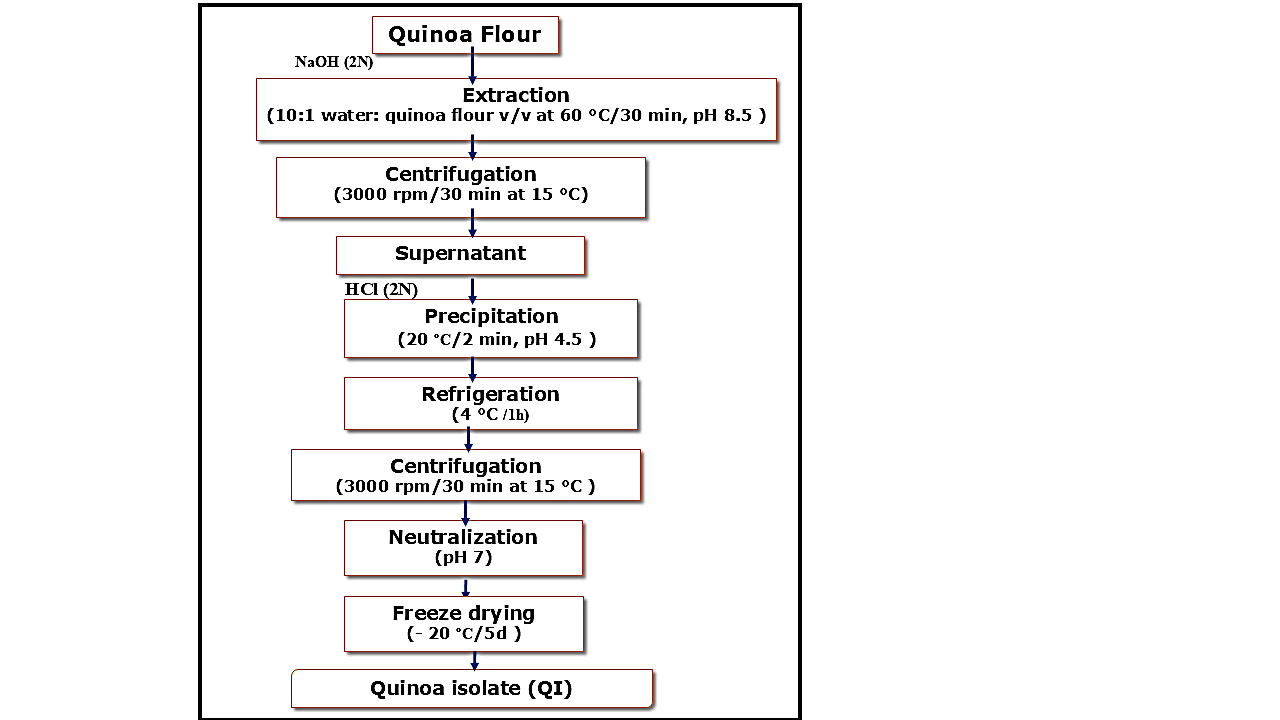


**Fig. SM1. Quinoa Protein Isolate (QPI) preparation**

### Spirulina extraction (SE)

### Spirulina ethanol extract was obtained using 80% ethanol according to Wang [8], with some modifications. The process involved mixing 100g of fine grounded, dried spirulina with 1L of 80% ethanol in a sealed glass container. This mixture was then heated in an 80°C water bath for 4 h. Following the heating period, the mixture underwent centrifugation at 2000 rpm/ 15 min to separate the liquid extract from the solid cellular debris. The extract was then decanted and subjected to a rotary evaporator at 50°C at 120 rpm under a vacuum of 1 bar to evaporate the ethanol, yielding a concentrated, dry extract.

### Phenolic Compounds Profile (HPLC)

High-performance liquid chromatography (HPLC) was performed using an Agilent 1260 system to analyze the samples. Compounds were separated on a Zorbax Eclipse Plus C8 column (4.6 mm x 250 mm, particle size: 5 μm). The mobile phase was a mixture of water (A) and acetonitrile containing 0.05% trifluoroacetic acid (B), flowing at 0.9 mL per minute. The separation process was optimized through a specific gradient elution protocol that involved varying the ratios of A and B over time. Detection occurred at a wavelength of 280 nm with a multi-wavelength detector. Each sample was injected with a volume of 5 μL, and the column temperature was consistently maintained at 40°C throughout the analysis [13].

### Spirulina extract encapsulation

The quinoa protein isolate (QPI) solution was prepared by dissolving QPI in a 75 mM Tris-HCl solution and adjusting the pH to 9.5 ± 0.1 with 0.5 M NaOH. The solution was then stirred at 25^o^C. Following centrifugation of the QPI solution at 8000 rpm/ 20 min, the resulting supernatant was subjected to dilution to yield a QPI stock solution with a concentration of 10 mg/mL through quantification via the Bradford method, as described by [9]. Subsequently, different concentrations of spirulina extract (SE) 0.2%, 0.4%, and 0.6% (w/v) were incorporated into the protein solution, with protein-polyphenol ratios of 1:0.2, 1:0.4, and 1:0.6, respectively. The same procedure was used to prepare a control sample, a QPI solution without SE[10].

### Characterization of QPI-NPS by DLS

The QPI, QPI-SE 0.2, QPI-SE 0.4, and QPI-SE 0.6 nanoparticles, were appropriately diluted and subjected to dynamic light scattering (DLS) analysis using a Nano-ZS90 instrument (Malvern, UK) to determine their respective particle size, polydispersity index (PDI), and zeta potential. The experiments were carried out in triplicate. Before determination, every sample of nano micelle was brought to an equilibrium state at a temperature of 25 ◦C [10].

Encapsulation efficiency (EE)

The SPE-QPI nanoparticles were subjected to ultra-sonication for 30 min after being mixed with absolute ethanol in a proportion of 1:9 (v/v) to facilitate the extraction of hydrophobic compounds. Following centrifugation at 3000 rpm/ 10 min, the sediments were subjected to a similar extraction process. The liquid portion, known as the supernatant, was gathered and consolidated. The solid phase extract (SPE) concentration was determined using a UV spectrophotometer (SP-UV 500DB, Spectrum Instruments, Canada) at a wavelength of 765 nm. The outcomes were denoted in mg of gallic acid equivalent (GAE) per g of spirulina extract powder (mg GAE/g). A regression analysis established a calibration curve using pure gallic acid at varying concentrations ranging from 0.155 to 0.180 mg/mL. The obtained regression coefficient was 0.9989 [30].

Microstructural Analysis via Transmission Electron Microscopy (TEM)

The microstructure of the nanoparticles was examined using Transmission Electron Microscopy (TEM). To prepare samples for TEM imaging, they were first diluted with ultra-pure water to achieve a concentration of 0.01%. A small volume of 10 μL of the diluted sample was then carefully applied to a carbon-coated copper grid and allowed to air dry. Subsequently, the prepared grids were analyzed using a JEOL JEM-1400 plus TEM, operating at 100 kV, with a magnification level of 200,000× employed for imaging [13]

Preparation of functional set yoghurt

Raw buffalo milk was utilized to prepare functional set yoghurt, which was divided into four groups: a control group (C) consisted of plain yoghurt, and three treatment groups designated as Treatment 1 (T1) was fortified with free spirulina extract (SE) at a concentration of 400 mg, Treatment 2 (T2) was fortified with 2000 mg of spirulina powder, and Treatment 3 (T3) was enhanced with 1400 mg of nano-encapsulated SE, specifically utilizing 0.4 SE-QPI nanoparticles (NPs). The selection of the 0.4 SE-QPI NPs formulation was based on the results of characterization studies that evaluated the polydispersity index, particle size, surface charge, and encapsulation efficiency (refer to Table 4). Each yoghurt sample underwent a heat treatment process, wherein the milk was heated to a temperature of 85-90°C for a duration of 5 minutes. Following the heat treatment, the samples were rapidly cooled to 42°C. At this stage, a 3% inoculation of a starter culture was introduced to each sample, along with a flavoring of 0.75% pistachio essence. The samples were subsequently incubated at 42°C for 6 hours, allowing for the development of a firm curd. After incubation, the prepared yoghurts were stored at a temperature of 5 ± 1°C, in preparation for subsequent analyses[8].

Physicochemical properties of set yoghurt

The pH of the yoghurt products was measured using a pH meter (model IQ 240, I.Q. Scientific Instruments Inc., San Diego, CA) equipped with automatic temperature compensation (A.T.C.) probe. Acidity was assessed following the [35]guidelines.

### Evaluation of antioxidant potentials

The sample capacity to scavenge free radicals was evaluated using DPPH (2,2-diphenyl-1-picrylhydrazyl) as described by [12]. A 0.1 mM solution of DPPH was prepared initially. Following this, 130 μL of the sample was mixed with 2000 μL of the solution. This mixture was kept in the dark for 30 minutes, after which the absorbance was measured at 510 nm using a Jasco V-630 spectrophotometer (USA). A control sample was prepared similarly, substituting the extract with ethanol, while methanol served as an additional control. The percentage of scavenging was calculated using the formula:

$DPPH Scavenging\%=\frac{A control -A sample}{A control} \times100 \ldots\ldots..$ 1

The total phenolic content of both the SE and the encapsulated SEE was assessed using the Folin-Ciocalteu method. In this procedure, 100 µL of the sample was mixed with diluted Folin-Ciocalteu reagent and allowed to react for 1 min. Subsequently, sodium carbonate solution was added, and the mixture was diluted to a final volume of 10 mL with ultra-pure water. After 2h of incubation in the dark, the absorbance of the solution was measured at 750 nm using a UV-Vis spectrophotometer. A standard curve of gallic acid (1-500 mg) was used to quantify the TPC, which was expressed in mg of gallic acid equivalents per gram of dry weight [12].

The total flavonoid content in the extracts was determined following the method established by [12]. In this procedure, 100 μL of 99% diethylene glycol and 100 μL of 1 N NaOH were added to 1 mL of each extract, and the mixture was stirred. The solution was allowed to react at 37 °C for one hour in a water bath. Absorbance was measured at 420 nm using a Jasco V-630 spectrophotometer (USA). Catechin was used as the reference compound, and the results were expressed as milligrams of catechin equivalents (CAE) per g of extract.

Viscosity

The Bohlin coaxial cylinder viscometer (Bohlin Instrument Inc., Sweden) was connected to a workstation with V88 viscometer programming software to assess the apparent viscosity of fresh yoghurt samples and at the end of storage period 21 days. The yoghurt sample cup was placed in the viscometer probe; model C30, and viscosity readings were recorded at 20 ± 2°C in the upward move across shear rates from 19 to 1236 S-1[13].

Sensory evaluation

In a controlled environment, sensory attributes of yoghurt samples served at 5°C in coded white cups were evaluated by 18 panelists aged between 27 to 51 years, from the Dairy Department, Food Industries and Nutrition Research Institute, the National Research Centre. The criteria for selection depended on their experience and background related to yoghurt products, and they were instructed to rinse their mouths with water between samples. The evaluation was carried out in standardized booths under artificial white light and at room temperature. Panelists were instructed to evaluate the yoghurt with respect to their degree of acceptance as follows; flavor (10 points), body & texture (10 points), color (10 points) and acceptability (10 points) as described by [13].

Statistical Analysis

All data are presented as mean ± standard deviation (SD). Statistical analyses were conducted using one-way analysis of variance (ANOVA) followed by Duncan’s test applying Co-Stat software (version 8). Statistical significance between groups was established at p ≤ 0.05. Data visualization was performed using GraphPad PRISM (Version 8.0.1, GraphPad Software, San Diego, CA, USA).

Table SM1. Phenolic compounds profile of spirulina and quinoa (HPLC)

| **Phenolic compounds** | **Spirulina**(µg/g) | **Quinoa** (µg/g) |
| --- | --- | --- |
| Gallic acid | 201.84 | 25.98 |
| Chlorogenic acid | 138.73 | 165.64 |
| Catechin | 41.62 | ND |
| Methyl gallate | 21.44 | 6.47 |
| Caffeic acid | ND | 14.17 |
| Syringic acid | ND | 6.21 |
| Pyro catechol | ND | ND |
| Rutin | ND | 7.14 |
| Ellagic acid | ND | ND |
| Coumaric acid | ND | 3.61 |
| Vanillin | ND | ND |
| Ferulic acid | 5.04 | 4.65 |
| Naringenin | 31.15 | 39.39 |
| Daidzein | 7.97 | 12.49 |
| Quercetin | 29.92 | ND |
| Cinnamic acid | 3.42 | 50.06 |
| Apigenin | ND | ND |
| Kaempferol | 7.19 | ND |
| Hesperetin | ND | ND |

| 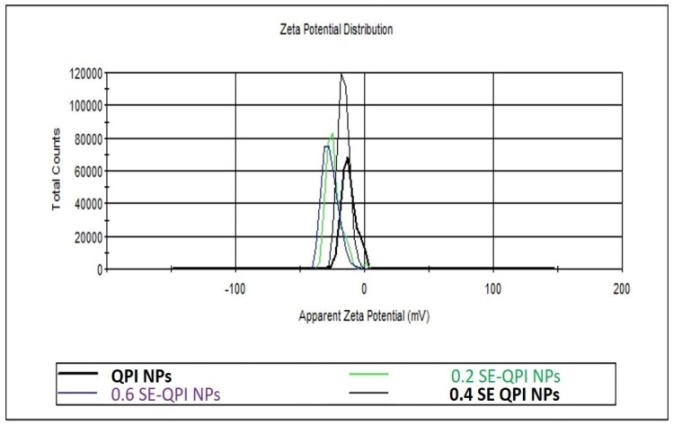 |  |
| --- | --- |

**Fig. SM2. Dynamic light scattering (DLS) and Zeta potential distribution of QPI-NPs and different concentrations of spirulina extract (SE) loaded in 1 g quinoa protein isolate nanoparticles (QPI-NPs) (0.2, 0.4, 0.6)**

- QPI NPs: quinoa protein isolate nanoparticles; 0.2 SE-QPI NPs: 0.2 g spirulina extract loaded in 1 g quinoa protein isolate nanoparticles; 0.4 SE-QPI NPs: 0.4 g spirulina extract loaded in 1 g quinoa protein isolate nanoparticles; 0.6 SE-QPI NPs: 0.6 g spirulina extract loaded in 1 g quinoa protein isolate nanoparticles.

| **a**  **a**  **b**  **a**  **a**  **ab**  **b**  **a**  **b**  **b**  **A**  **a**  **a**  **a**  **a**  **c**  **c** | **b**  **b**  **b**  **b**  **ab**  **ab**  **ab**  **ab**  **a**  **a**  **a**  **a**  **a**  **a**  **a**  **a**  **B** |
| --- | --- |

**Fig.SM3. pH (A) and Acidity (B) of functional fortified set**

- Values are represented as means ± SD.
- Different letters indicate the differences between samples at the same time interval (Fresh, 7, 14, 21 days) (*P*<0.05).
- **C:** control plain set yoghurt prepared from buffalo milk; **T1:** yoghurt fortified with free spirulina extract (SE) at a concentration of 400 mg; **T2:** yoghurt fortified with 2 g spirulina powder; **T3:** yoghurt fortified with 0.4 g spirulina extract nanoencapsulated which containing 400 mg spirulina extract loaded in 1000 mg quinoa protein isolate.

**
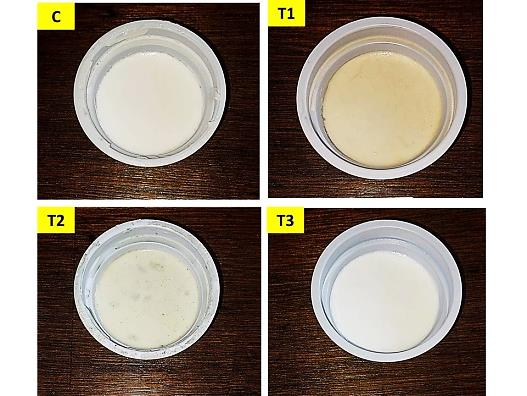
**

**Fig.SM3. Functional fortified set yoghurt samples**

C: control plain set yoghurt prepared from buffalo milk; T1: yoghurt fortified with free spirulina extract (SE) at a concentration of 400 mg; T2: yoghurt fortified with 2 g spirulina powder; T3: yoghurt fortified with 0.4 g spirulina extract nanoencapsulated which containing 400 mg spirulina extract loaded in 1000 mg quinoa protein isolate.

### Materials and methods

Sweet quinoa seeds Cv. Masr1 were obtained from Agricultural Research Center (ARC), Giza, Egypt. Spirulina powder was obtained from the culture collection of algae at Chad. Buffalo milk was obtained from Faculty of Agriculture, Cairo University, Giza, Egypt. Yoghurt starter (containing mixture of *Lactobacillus delbreukii* subsp *bulgaricus* and *streptococcus thermophilus* (1:1)) was obtained from Christen Hansen Laboratories, Copenhagen, Denmark, and kept at –18°C, until activated before use in sterilized reconstituted skim milk powder (SMP) obtained from Dairy America, Inc., California, USA, composed of 34% protein, 51% lactose, 1.2% fat, 8.2% minerals, and 4% moisture). Chemical materials used were purchased from Sigma Aldrich, Merck (Sigma Aldrich, Merck St. Louis, MO, USA). All reagents and solvents used were analytical grade.

**Preparation quinoa flour and protein isolate (QF & PI)**

Quinoa seeds were sieved 250-micrometer opening to foreign materials, soaked in water 12h/ 20^o^C, then washed till saponin removal and dried at 45∘C/12h [29]. Seeds then were dried to 12% moisture at 120°C/20 min, cooled at room temperature, grounded, sieved with a mesh size of 20.0 (840 μm), packed in polythene plastic bags and stored at -20 °C.

For quinoa protein isolate (QPI) preparation; the quinoa flour was defatted according to Folch [28]. Quinoa protein isolate was prepared according to El-Sohaimy [29]. Fifty grams of defatted quinoa flour were suspended in 1000 mL deionized distilled water (1:10 v/v), at pH 8.5, stirred for 1 h at 60oC/30 min, centrifuged at 3000 rpm at 15°C/ 30 min (model K241R, Pro-Research, Centurion Scientific Ltd, UK). The pH was acidified to 4.5, refrigerated at 4oC/1h for precipitation, centrifuged at 3000 rpm at 15°C/ 30 min. The protein isolate was the neutralized and freeze dried at -20oC.The obtained quinoa flour was defatted three times with chloroform: methanol (2:1), 1:10 w/v with shaking for 2 h according to Folch [28]. Quinoa protein isolate was prepared according to El-Sohaimy [29]. Fifty grams of defatted quinoa flour were suspended in 1000 mL deionized distilled water (1:10 v/v), and the pH was adjusted to 8.5 using 2N (NaOH) in order to facilitate quinoa protein solubility for higher yield of protein isolate. The suspension was stirred for 1 h while maintaining the pH at the determined value to reach the maximum level of solubility at 60^o^C/30 min. The mixture was centrifuged at 3000 rpm at 15°C/ 30 min by a high-speed cooling centrifuge (model K241R, Pro-Research, Centurion Scientific Ltd, UK). The supernatant was then adjusted to pH 4.5 with HCl 2N which is the isoelectric point of most proteins, refrigerated to 4^o^C/1h to facilitate protein precipitation and the centrifuged at 3000 rpm at 15°C/ 30 min for protein separation. The protein isolate was then neutralized with 2N (NaOH), freeze dried -20oC/ 5days as illustrated in (**Fig SM1).**


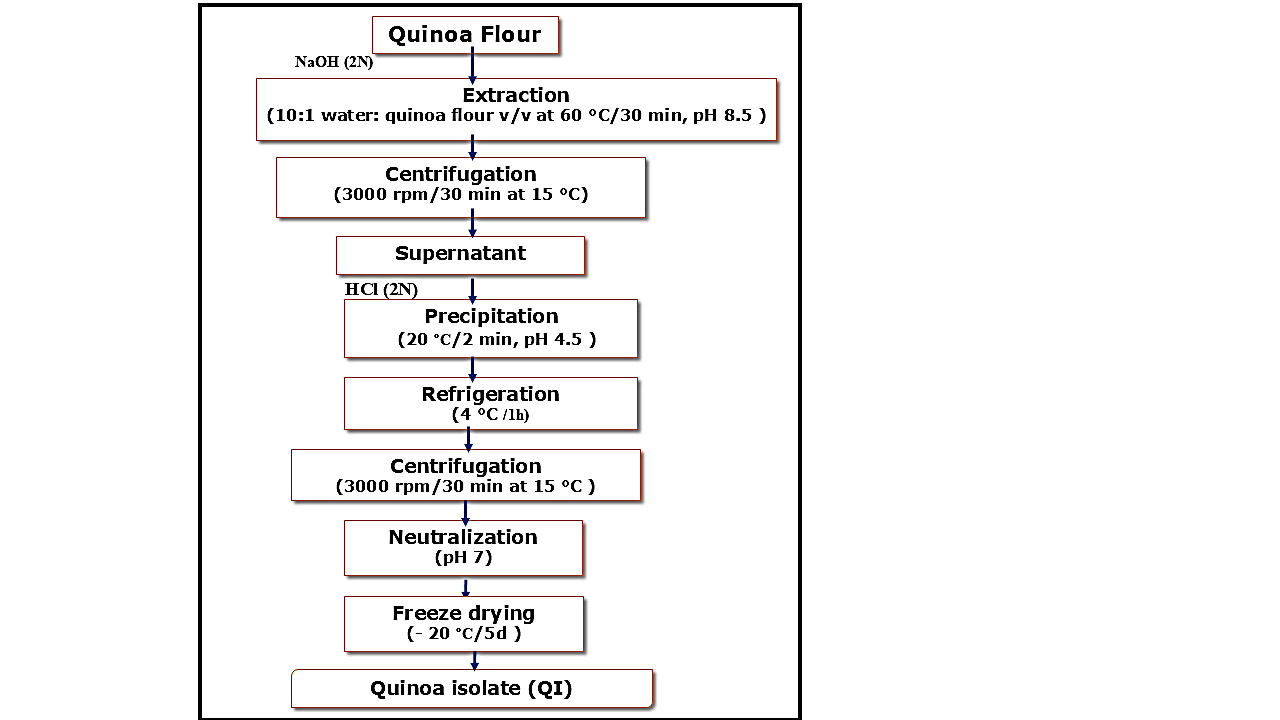


**Fig. SM1. Quinoa Protein Isolate (QPI) preparation**

### Spirulina extraction (SE)

### Spirulina ethanol extract was obtained using 80% ethanol according to Wang [8], with some modifications. The process involved mixing 100g of fine grounded, dried spirulina with 1L of 80% ethanol in a sealed glass container. This mixture was then heated in an 80°C water bath for 4 h. Following the heating period, the mixture underwent centrifugation at 2000 rpm/ 15 min to separate the liquid extract from the solid cellular debris. The extract was then decanted and subjected to a rotary evaporator at 50°C at 120 rpm under a vacuum of 1 bar to evaporate the ethanol, yielding a concentrated, dry extract.

### Phenolic Compounds Profile (HPLC)

High-performance liquid chromatography (HPLC) was performed using an Agilent 1260 system to analyze the samples. Compounds were separated on a Zorbax Eclipse Plus C8 column (4.6 mm x 250 mm, particle size: 5 μm). The mobile phase was a mixture of water (A) and acetonitrile containing 0.05% trifluoroacetic acid (B), flowing at 0.9 mL per minute. The separation process was optimized through a specific gradient elution protocol that involved varying the ratios of A and B over time. Detection occurred at a wavelength of 280 nm with a multi-wavelength detector. Each sample was injected with a volume of 5 μL, and the column temperature was consistently maintained at 40°C throughout the analysis [13].

### Spirulina extract encapsulation

The quinoa protein isolate (QPI) solution was prepared by dissolving QPI in a 75 mM Tris-HCl solution and adjusting the pH to 9.5 ± 0.1 with 0.5 M NaOH. The solution was then stirred at 25^o^C. Following centrifugation of the QPI solution at 8000 rpm/ 20 min, the resulting supernatant was subjected to dilution to yield a QPI stock solution with a concentration of 10 mg/mL through quantification via the Bradford method, as described by [9]. Subsequently, different concentrations of spirulina extract (SE) 0.2%, 0.4%, and 0.6% (w/v) were incorporated into the protein solution, with protein-polyphenol ratios of 1:0.2, 1:0.4, and 1:0.6, respectively. The same procedure was used to prepare a control sample, a QPI solution without SE[10].

### Characterization of QPI-NPS by DLS

The QPI, QPI-SE 0.2, QPI-SE 0.4, and QPI-SE 0.6 nanoparticles, were appropriately diluted and subjected to dynamic light scattering (DLS) analysis using a Nano-ZS90 instrument (Malvern, UK) to determine their respective particle size, polydispersity index (PDI), and zeta potential. The experiments were carried out in triplicate. Before determination, every sample of nano micelle was brought to an equilibrium state at a temperature of 25 ◦C [10].

Encapsulation efficiency (EE)

The SPE-QPI nanoparticles were subjected to ultra-sonication for 30 min after being mixed with absolute ethanol in a proportion of 1:9 (v/v) to facilitate the extraction of hydrophobic compounds. Following centrifugation at 3000 rpm/ 10 min, the sediments were subjected to a similar extraction process. The liquid portion, known as the supernatant, was gathered and consolidated. The solid phase extract (SPE) concentration was determined using a UV spectrophotometer (SP-UV 500DB, Spectrum Instruments, Canada) at a wavelength of 765 nm. The outcomes were denoted in mg of gallic acid equivalent (GAE) per g of spirulina extract powder (mg GAE/g). A regression analysis established a calibration curve using pure gallic acid at varying concentrations ranging from 0.155 to 0.180 mg/mL. The obtained regression coefficient was 0.9989 [30].

Microstructural Analysis via Transmission Electron Microscopy (TEM)

The microstructure of the nanoparticles was examined using Transmission Electron Microscopy (TEM). To prepare samples for TEM imaging, they were first diluted with ultra-pure water to achieve a concentration of 0.01%. A small volume of 10 μL of the diluted sample was then carefully applied to a carbon-coated copper grid and allowed to air dry. Subsequently, the prepared grids were analyzed using a JEOL JEM-1400 plus TEM, operating at 100 kV, with a magnification level of 200,000× employed for imaging [13]

Preparation of functional set yoghurt

Raw buffalo milk was utilized to prepare functional set yoghurt, which was divided into four groups: a control group (C) consisted of plain yoghurt, and three treatment groups designated as Treatment 1 (T1) was fortified with free spirulina extract (SE) at a concentration of 400 mg, Treatment 2 (T2) was fortified with 2000 mg of spirulina powder, and Treatment 3 (T3) was enhanced with 1400 mg of nano-encapsulated SE, specifically utilizing 0.4 SE-QPI nanoparticles (NPs). The selection of the 0.4 SE-QPI NPs formulation was based on the results of characterization studies that evaluated the polydispersity index, particle size, surface charge, and encapsulation efficiency (refer to Table 4). Each yoghurt sample underwent a heat treatment process, wherein the milk was heated to a temperature of 85-90°C for a duration of 5 minutes. Following the heat treatment, the samples were rapidly cooled to 42°C. At this stage, a 3% inoculation of a starter culture was introduced to each sample, along with a flavoring of 0.75% pistachio essence. The samples were subsequently incubated at 42°C for 6 hours, allowing for the development of a firm curd. After incubation, the prepared yoghurts were stored at a temperature of 5 ± 1°C, in preparation for subsequent analyses[8].

Physicochemical properties of set yoghurt

The pH of the yoghurt products was measured using a pH meter (model IQ 240, I.Q. Scientific Instruments Inc., San Diego, CA) equipped with automatic temperature compensation (A.T.C.) probe. Acidity was assessed following the [11]guidelines.

### Evaluation of antioxidant potentials

The sample capacity to scavenge free radicals was evaluated using DPPH (2,2-diphenyl-1-picrylhydrazyl) as described by [12]. A 0.1 mM solution of DPPH was prepared initially. Following this, 130 μL of the sample was mixed with 2000 μL of the solution. This mixture was kept in the dark for 30 minutes, after which the absorbance was measured at 510 nm using a Jasco V-630 spectrophotometer (USA). A control sample was prepared similarly, substituting the extract with ethanol, while methanol served as an additional control. The percentage of scavenging was calculated using the formula:

$DPPH Scavenging\%=\frac{A control -A sample}{A control} \times100 \ldots\ldots..$ 1

The total phenolic content of both the SE and the encapsulated SEE was assessed using the Folin-Ciocalteu method. In this procedure, 100 µL of the sample was mixed with diluted Folin-Ciocalteu reagent and allowed to react for 1 min. Subsequently, sodium carbonate solution was added, and the mixture was diluted to a final volume of 10 mL with ultra-pure water. After 2h of incubation in the dark, the absorbance of the solution was measured at 750 nm using a UV-Vis spectrophotometer. A standard curve of gallic acid (1-500 mg) was used to quantify the TPC, which was expressed in mg of gallic acid equivalents per gram of dry weight [12].

The total flavonoid content in the extracts was determined following the method established by [12]. In this procedure, 100 μL of 99% diethylene glycol and 100 μL of 1 N NaOH were added to 1 mL of each extract, and the mixture was stirred. The solution was allowed to react at 37 °C for one hour in a water bath. Absorbance was measured at 420 nm using a Jasco V-630 spectrophotometer (USA). Catechin was used as the reference compound, and the results were expressed as milligrams of catechin equivalents (CAE) per g of extract.

Viscosity

The Bohlin coaxial cylinder viscometer (Bohlin Instrument Inc., Sweden) was connected to a workstation with V88 viscometer programming software to assess the apparent viscosity of fresh yoghurt samples and at the end of storage period 21 days. The yoghurt sample cup was placed in the viscometer probe; model C30, and viscosity readings were recorded at 20 ± 2°C in the upward move across shear rates from 19 to 1236 S-1[13].

Sensory evaluation

In a controlled environment, sensory attributes of yoghurt samples served at 5°C in coded white cups were evaluated by 18 panelists aged between 27 to 51 years, from the Dairy Department, Food Industries and Nutrition Research Institute, the National Research Centre. The criteria for selection depended on their experience and background related to yoghurt products, and they were instructed to rinse their mouths with water between samples. The evaluation was carried out in standardized booths under artificial white light and at room temperature. Panelists were instructed to evaluate the yoghurt with respect to their degree of acceptance as follows; flavor (10 points), body & texture (10 points), color (10 points) and acceptability (10 points) as described by [13].

Statistical Analysis

All data are presented as mean ± standard deviation (SD). Statistical analyses were conducted using one-way analysis of variance (ANOVA) followed by Duncan’s test applying Co-Stat software (version 8). Statistical significance between groups was established at p ≤ 0.05. Data visualization was performed using GraphPad PRISM (Version 8.0.1, GraphPad Software, San Diego, CA, USA).

Table SM1. Phenolic compounds profile of spirulina and quinoa (HPLC)

| **Phenolic compounds** | **Spirulina**(µg/g) | **Quinoa** (µg/g) |
| --- | --- | --- |
| Gallic acid | 201.84 | 25.98 |
| Chlorogenic acid | 138.73 | 165.64 |
| Catechin | 41.62 | ND |
| Methyl gallate | 21.44 | 6.47 |
| Caffeic acid | ND | 14.17 |
| Syringic acid | ND | 6.21 |
| Pyro catechol | ND | ND |
| Rutin | ND | 7.14 |
| Ellagic acid | ND | ND |
| Coumaric acid | ND | 3.61 |
| Vanillin | ND | ND |
| Ferulic acid | 5.04 | 4.65 |
| Naringenin | 31.15 | 39.39 |
| Daidzein | 7.97 | 12.49 |
| Quercetin | 29.92 | ND |
| Cinnamic acid | 3.42 | 50.06 |
| Apigenin | ND | ND |
| Kaempferol | 7.19 | ND |
| Hesperetin | ND | ND |

| 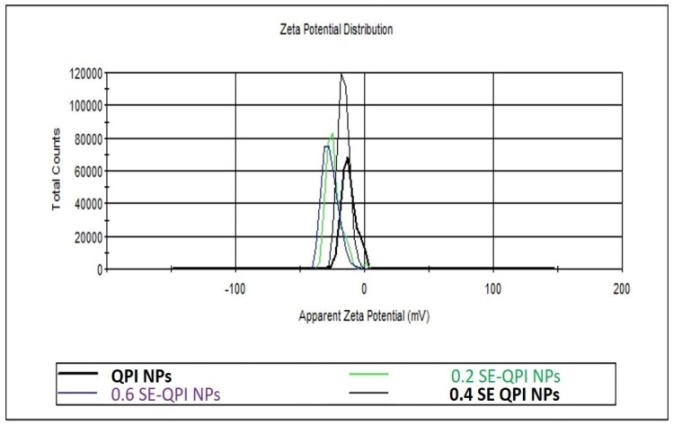 |  |
| --- | --- |

**Fig. SM2. Dynamic light scattering (DLS) and Zeta potential distribution of QPI-NPs and different concentrations of spirulina extract (SE) loaded in 1 g quinoa protein isolate nanoparticles (QPI-NPs) (0.2, 0.4, 0.6)**

- QPI NPs: quinoa protein isolate nanoparticles; 0.2 SE-QPI NPs: 0.2 g spirulina extract loaded in 1 g quinoa protein isolate nanoparticles; 0.4 SE-QPI NPs: 0.4 g spirulina extract loaded in 1 g quinoa protein isolate nanoparticles; 0.6 SE-QPI NPs: 0.6 g spirulina extract loaded in 1 g quinoa protein isolate nanoparticles.

| **a**  **a**  **b**  **a**  **a**  **ab**  **b**  **a**  **b**  **b**  **A**  **a**  **a**  **a**  **a**  **c**  **c** | **b**  **b**  **b**  **b**  **ab**  **ab**  **ab**  **ab**  **a**  **a**  **a**  **a**  **a**  **a**  **a**  **a**  **B** |
| --- | --- |

**Fig.SM3. pH (A) and Acidity (B) of functional fortified set**

- Values are represented as means ± SD.
- Different letters indicate the differences between samples at the same time interval (Fresh, 7, 14, 21 days) (*P*<0.05).
- **C:** control plain set yoghurt prepared from buffalo milk; **T1:** yoghurt fortified with free spirulina extract (SE) at a concentration of 400 mg; **T2:** yoghurt fortified with 2 g spirulina powder; **T3:** yoghurt fortified with 0.4 g spirulina extract nanoencapsulated which containing 400 mg spirulina extract loaded in 1000 mg quinoa protein isolate.

**
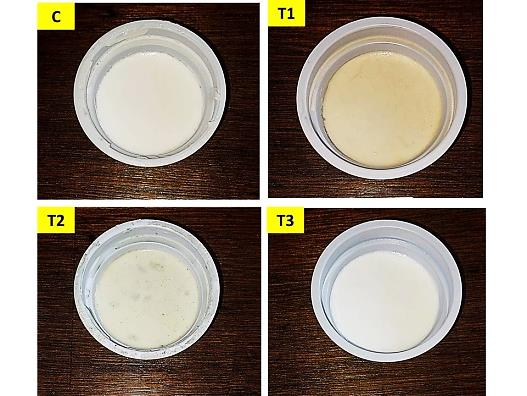
**

**Fig.SM3. Functional fortified set yoghurt samples**

C: control plain set yoghurt prepared from buffalo milk; T1: yoghurt fortified with free spirulina extract (SE) at a concentration of 400 mg; T2: yoghurt fortified with 2 g spirulina powder; T3: yoghurt fortified with 0.4 g spirulina extract nanoencapsulated which containing 400 mg spirulina extract loaded in 1000 mg quinoa protein isolate.
